# COVID‐19 and a Pulmonary Abscess in a 2‐Month Infant: A Case Report

**DOI:** 10.1002/rcr2.70179

**Published:** 2025-05-13

**Authors:** Rana Tafrishi, Abbas Boskabadi, Ahmad Mohammadipour, Seyed Javad Seyedi

**Affiliations:** ^1^ Department of Allergy and Immunology, Ghaem Hospital Mashhad University of Medical Sciences Mashhad Iran; ^2^ Department of Pediatrics, Faculty of Medicine Mashhad University of Medical Sciences Mashhad Iran; ^3^ Department of Pediatric Surgery, Akbar Hospital Mashhad University of Medical Sciences Mashhad Iran

**Keywords:** complication, COVID‐19, infant, infection, lung abscess

## Abstract

Severe acute respiratory syndrome coronavirus 2 (SARS‐CoV‐2) is an acute respiratory syndrome that has had a significant global impact. Some studies reported that SARS‐CoV‐2 infected adults, especially intubated individuals, are at risk of lung abscess, but a few studies show lung abscess in infants. We present a 2‐month‐old infant with a lung abscess and positive SARS‐CoV‐2 PCR.

## Graphical abstract

This study presents a 2‐month‐old infant girl with a lung abscess with positive SARS‐CoV‐2 PCR.

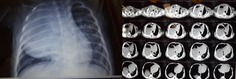

## 1 Introduction

Lung abscesses are uncommon in childhood and extremely rare in early infancy and neonates, with an estimated incidence of 7 cases per 100,000 hospital admissions annually. Moreover, neonatal lung abscesses typically have a polymicrobial origin. Additionally, risk factors in neonates include prematurity, mechanical ventilation, congenital lung anomalies, and aspiration [1].

In December 2019, a cluster of patients with pneumonia and other respiratory symptoms emerged in Wuhan province, Hubei, China. A coronavirus caused this pneumonia and led to severe acute respiratory syndrome (SARS). The world health organisation (WHO) named the severe acute respiratory syndrome coronavirus 2 (SARS‐CoV‐2). More than 2 years after its first detection in China, the disease has devastated populations worldwide. The virus continues to spread despite stringent interdisciplinary measures. SARS‐CoV‐2 has a wide range of symptoms; mild symptoms include: fever, fatigue, anorexia, myalgia, and diarrhoea, and severe symptoms include: dyspnea, hypoxemia, and rapid progression to respiratory failure and death. SARS‐CoV‐2 has also been reported to have further medical complications [2]. Viral infection may increase the risk of bacterial infection by several mechanisms [3]. This study presents a 2‐month‐old girl infant with a lung abscess with positive SARS‐CoV‐2 PCR.

## 2 Case Report

A two‐month‐old girl presented to the emergency department with cyanosis and a 2‐week history of fever and cough. One week before, she was resuscitated and intubated in another hospital because of sudden respiratory distress and cyanosis. She was extubated after 4 days. In her past medical history, her gestational age was 34 weeks, she was born from a twin pregnancy by caesarean section, and she was hospitalised in NICU in the first week of life due to prematurity and jaundice. No pulmonary anomaly was reported in her chest X ray.

**FIGURE 1:**
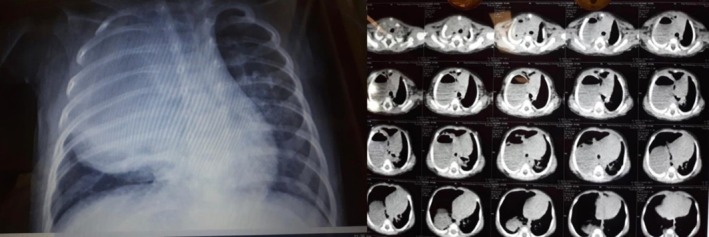
Chest x‐ray (Left) and chest CT scan (Right) of patient showing abscess in right upper lobe (before surgical operation).

At the initial examination, the patient had mild retraction and respiratory distress (RR = 66), an SPO_2_ of 77% without oxygenation and 94% with an oxygen mask, and a temperature of 37.2°C (Table 1). The patient was admitted, and a chest x‐ray showed a mass‐like lesion in the right upper lobe (Figure 1).

**TABLE 1:** Laboratory findings of patient.

Variables			Value	Normal range
ABG	PH		7.43	7.35–7.45
	PCO_2_ (mmHg)		26	35–45 mmHg
	HCO_3_ (mEq/L)		20	22–26 mEq/L
	PO_2_ (mmHg)		65	80–100 mmHg
CBC	White blood cell	(10^3^/mL)	14.3	6–16 (10^3^/mL)
		Poly morphonuclear (%)	25%	40%–70%
		Lymphocytes (%)	64%	20%–40%
	Haemoglobin (g/dL)		12.8	11.1–14.1 (g/dL)
	Platelet (10^3^/mcL)		493	200–550 (10^3^/mcl)
Lactate dehydrogenase (U/L)			678	140–280 U/L
Ferritin (ngr/mL)			295	Males: 30–400 ng/mL Females: 15–150 ng/mL
Alpha‐fetoprotein (IU/mL)			> 300	Less than 10 IU/mL
Β hCG			< 1	Less than 5 IU/mL
C‐reactive protein (mg/L)			31	0–5 (mg/L)
Erythrocyte sedimentation rate (mm)			44	Males: less than 15 mm/h Females: less than 20 mm/h
Uric acid (mg/dL)			1.8	Males: 3.5–7.2 mg/dL Females: 2.6–6.0 mg/dL
Blood culture			Negative	Negative (Normal)

Ultrasound of the chest showed a heterogeneous mass of 32 × 56 mm in the upper lobe of the right lung. A subsequent high‐resolution computed tomography (HRCT) was performed, showing evidence favouring a lung abscess in the upper lobe of the right lung (Figure 1). Lung abscess may be a primary infection or as a complication secondary to inadequate antibiotic therapy and superinfection. Normal echocardiography and abdominal ultrasound were reported. A COVID‐19 PCR test was performed for the patient as a routine during the pandemic, which was positive. COVID‐19 PCR was also positive for both parents. The influenza PCR test was negative.

**FIGURE 2:**
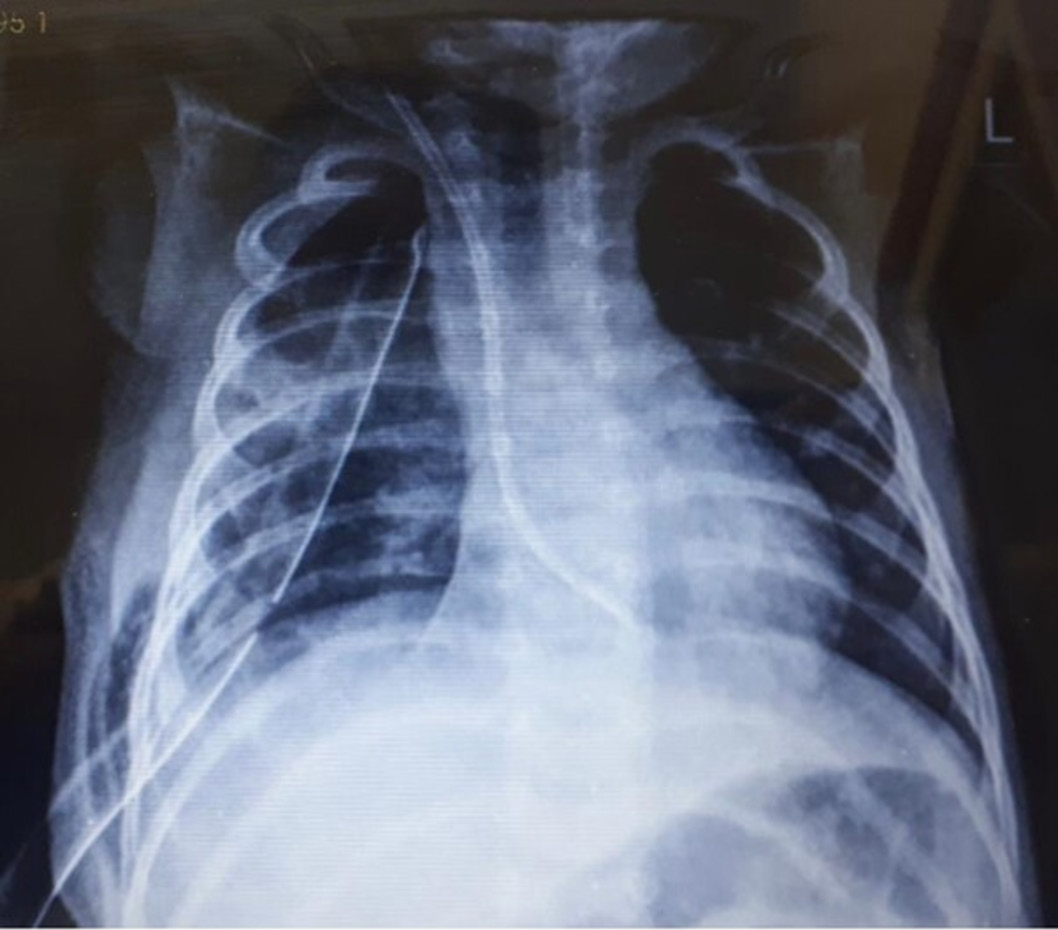
Chest x‐ray of patient after surgical operation showing complete resolution of abscess.

After being transferred to the operating room, the patient underwent thoracotomy and lobectomy of the right upper lobe, and the patient's abscess was drained (Figure 2). Meropenem and vancomycin were also initiated and continued for 2 weeks. There was no positive culture of abscess pus for bacterial and mycobacterial organisms, probably due to previous antibiotic consumption. The discharged secretions collected in the chest bottle appeared purulent and cloudy, with a thick consistency and a yellowish‐green colour. The pathologist reported a 4 cm cyst in the right upper lobe, acute pneumonia with ectatic and ulcerated bronchi with bronchiectasis. Alveolar collapse and subpleural haemorrhage were also seen. The patient was discharged after recovery and was followed for 1 year, during which no complications were seen, and the patient had a normal growth pattern. The immunologic profile was within normal limits.

## 3 Discussion

Lung abscesses can be classified as either primary (in previously healthy children) or secondary on the presence of predisposing causes including inherited lung diseases, such as congenital pulmonary airway malformation, cystic fibrosis, or primary ciliary dyskinesia, as well as in patients with immunocompromised conditions like immunodeficiencies [4].

Due to the interconnected modern world, we were facing one of the most challenging pandemics in the history of humanity. It is known that the disease is caused by the SARS‐CoV2 virus, which belongs to the more prominent coronavirus family, mainly a respiratory pathogen. Knowledge of its mode of transmission and the spectrum of clinical presentation is vital for a more practical approach to the condition [5].

The most common symptoms of SARS‐CoV‐2 are fever, dry cough, sore throat, dyspnea, fatigue, and myalgia. However, several cases of atypical presentations with diagnostic and management dilemmas have been reported in patients with SARS‐CoV‐2 [6, 7, 8].

During the clinical progression of COVID‐19, the likelihood of bacterial or fungal infections rises. These infections are categorised into co‐infections, which are identified at the time of COVID‐19 diagnosis, and secondary infections, which develop after the initial presentation of the disease. Since the initiation of the SARS‐CoV‐2 epidemic in China, bacterial and fungal infections have been described several times following SARS‐CoV‐2 pneumonia. Super‐infections such as fungal and bacterial infections can result from pulmonary cavitation, a complication of thrombosis, and pulmonary infarction due to thrombosis induced by COVID‐19 [9, 10, 11]. Harikrishnan et al. reported an adult female patient with positive PCR for SARS‐CoV‐2 and lung abscess [12, 13]. Another study reported delayed lung abscess in a patient 1 month after recovery.

A previous study reported that 9.3% of patients hospitalised with COVID‐19 were complicated by bacterial infection and increased to 43% when critical care was needed. Common pathogens were *Pseudomonas aeruginosa* , Klebsiella species, *Staphylococcus aureus* , *Escherichia coli* and *Stenotrophomonas maltophilia* [14].

The present case report suggested more caution about long‐term complications of COVID‐19 [8]. The susceptibility to SARS‐CoV‐2 infection of the patients with a history of Lung abscess is higher due to impairment of nonspecific immune elements, deterioration of vascular architecture, poor oxygenation in the post‐abscess scar region, and lifestyle factors [15]. It remains unknown whether these complications directly result from specific SARS‐CoV‐2 toxicity.

In conclusion, this case demonstrated that COVID‐19 can increase the risk of lung abscesses in infants. Therefore, we recommend that ill infants with a positive COVID‐19 PCR test and elevated CRP levels be evaluated for secondary bacterial infections, including lung abscesses. Late complications of COVID‐19, such as lung abscesses, should be considered and carefully monitored both during hospitalisation and after discharge.

## Author Contributions

Rana Tafrishi and Abbas Boskabadi drafted the initial manuscript and prepared the images. All authors revised the manuscript and approved the final version of the manuscript.

## Ethics Statement

The authors declare that written informed consent was obtained for the publication of this manuscript and accompanying images using the consent form provided by the Journal.

## Conflicts of Interest

The authors declare no conflicts of interest.

## Data Availability

The data that support the findings of this study are available on request from the corresponding author. The data are not publicly available due to privacy or ethical restrictions.
